# Multi-Allelic Mitochondrial DNA Deletions in an Adult Dog with Chronic Weakness, Exercise Intolerance and Lactic Acidemia

**DOI:** 10.3390/ani14131946

**Published:** 2024-06-30

**Authors:** G. Diane Shelton, James R. Mickelson, Steven G. Friedenberg, Jonah N. Cullen, Jaya M. Mehra, Ling T. Guo, Katie M. Minor

**Affiliations:** 1 Department of Pathology, School of Medicine, University of California San Diego, La Jolla, CA 92093-0709, USA; liguo@health.ucsd.edu; 2 Department of Veterinary and Biomedical Sciences, College of Veterinary Medicine, University of Minnesota, St. Paul, MN 55108, USA; micke001@umn.edu; 3 Department of Veterinary Clinical Sciences, College of Veterinary Medicine, University of Minnesota, St. Paul, MN 55108, USA; fried255@umn.edu (S.G.F.); cull0084@umn.edu (J.N.C.); minork@umn.edu (K.M.M.); 4 VCA Animal Care Center of Sonoma County, Rohnert Park, CA 94928, USA; jaya.mehra@vca.com

**Keywords:** canine, muscle, myopathy, whole-genome sequencing (WGS), variant analysis

## Abstract

**Simple Summary:**

Mitochondrial diseases in people are a heterogeneous group of genetically, biochemically, and clinically diverse disorders caused by defects in the metabolic pathways of oxidation phosphorylation (OXPHOS) and ATP production. While infrequently described in dogs, mitochondrial diseases may be more common than is currently known. Both the nuclear and mitochondrial genomes encode components of the enzyme complexes of the OXPHOS system, resulting in the genetic bases of mitochondrial diseases being transmitted in a Mendelian fashion, maternally, or as sporadic diseases. The pathogenicity is further complicated by heteroplasmy, with several different mitochondrial genomes existing within a given cell. A major challenge in the diagnosis of mitochondrial diseases is the clinical heterogeneity, with multi-system diseases affecting three or more organs and characteristically affecting tissues with high energy demands such as the central nervous system, skeletal muscle, eye and heart. Novel clinical syndromes have been reported in association with mitochondrial dysfunction and many patients can be misdiagnosed. The advent of next-generation sequencing has resulted in the discovery of new pathogenetic variants and has become the standard for the diagnosis of mitochondrial disease. Here, we describe an adult dog with a chronic myopathy associated with a mitochondrial deletion disorder.

**Abstract:**

(1) Background: An adult dog was presented to a board-certified veterinary neurologist for evaluation of chronic weakness, exercise intolerance and lactic acidemia. (2) Methods: A mitochondrial myopathy was diagnosed based on the histological and histochemical phenotype of numerous COX-negative muscle fibers. Whole-genome sequencing established the presence of multiple extended deletions in the mitochondrial DNA (mtDNA), with the highest prevalence between the 1–11 kb positions of the approximately 16 kb mitochondrial chromosome. Such findings are typically suggestive of an underlying nuclear genome variant affecting mitochondrial replication, repair, or metabolism. (3) Results: Numerous variants in the nuclear genome unique to the case were identified in the whole-genome sequence data, and one, the insertion of a *DYNLT1* retrogene, whose parent gene is a regulator of the mitochondrial voltage-dependent anion channel (VDAC), was considered a plausible causal variant. (4) Conclusions: Here, we add mitochondrial deletion disorders to the spectrum of myopathies affecting adult dogs.

## 1. Introduction

Mitochondrial diseases (mtD) in people are a heterogeneous group of genetically, biochemically, and clinically diverse disorders caused by defects in the metabolic pathways of oxidation phosphorylation (OXPHOS) and ATP production [1,2]. Both the nuclear and mitochondrial genomes encode components of the enzyme complexes of the OXPHOS system, resulting in the genetic bases of mtD being transmitted in a Mendelian fashion, maternally, or as sporadic diseases. The pathogenicity is further complicated by heteroplasmy, in which several different mitochondrial genomes exist within a given cell [3]. A major challenge in the diagnosis of mtD is the clinical heterogeneity of these disorders, with most patients having a multi-system disease with three or more organs affected, characteristically affecting tissues with high energy demands such as the central nervous system, skeletal muscle, eye and heart [4]. Novel clinical syndromes have been reported in association with mitochondrial dysfunction and many patients can be misdiagnosed. The advent of next-generation sequencing has resulted in the discovery of new pathogenetic variants and has become the standard for the diagnosis of mtDs [5].

Mitochondrial diseases can arise from mutations in both nuclear and mitochondrial genomes as most proteins involved in mitochondrial metabolism, and all those involved in mtDNA maintenance, are encoded in the nuclear DNA (nDNA) [6,7,8]. Mutations in more than 350 genes, both nuclear and mitochondrial, are currently known to cause primary mitochondrial disorders in people [5]. Those disorders caused by a large-scale mutation in the mtDNA include Kearns–Sayre syndrome [9,10], chronic progressive external ophthalmoplegia (CPEO) [9,11], myopathy, encephalopathy, lactic acidosis and stroke-like episodes (MELAS) [12,13,14], and myoclonic episodes with ragged-red fibers (MERRF) [15], among others. Cases with multiple mtDNA deletions (i.e., deletions of various sizes in the mtDNA) are usually associated with a nuclear gene defect involved in mtDNA maintenance which can provide guidance for analysis of variants detected by next-generation sequencing [3].

In dogs, variants in both nuclear and mitochondrial DNA have also been associated with four forms of encephalopathies and five forms of myopathies in various breeds. Encephalopathies include two different *SLC19A3* gene variants associated with subacute necrotizing encephalopathy or Leigh syndrome (OMIA#001097-9615), one in Alaskan Huskies [16] and the other in Yorkshire terriers [17]; a *cytochrome b* variant (OMIA#002684-9615) causing canine spongiform leukoencephalomyelopathy in Australian blue heelers and Shetland sheepdogs [18]; and a *NDUFS7* variant (OMIA#002840-9615) in a Jack Russell–Chihuahua mixed-breed dog, the most common gene associated with Leigh syndrome in people [19]. Nuclear gene variants associated with canine mitochondrial myopathies include a mutation in the *PDP1* gene (OMIA#001406-9615), encoding the phosphatase enzyme that activates the pyruvate dehydrogenase complex, in Clumber and Sussex spaniels [20]; a variant in the mitochondrial aspartate/glutamate carrier gene *SLC25A12* (OMIA#002294-9615) that causes an inflammatory myopathy in Dutch shepherd dogs [21]; and a variant in the *ACADVL* gene (OMIA# 002140-9615) encoding the very-long-chain acryl-CoA dehydrogenase associated with exercise-induced myopathy in German Hunting terriers [22]. A mitochondrial myopathy in Old English Sheepdogs associated with reduced mitochondrial mRNA and decreased cytochrome c oxidase activities in fibroblasts and skeletal muscle has been reported although a specific gene variant has not been identified [23]. Several other case reports have described mitochondrial myopathies in dogs based on histopathological and morphological changes, and biochemical activities, but these have also not yet been subjected to genetic evaluation [24]. A sensory ataxic neuropathy associated with a deletion in the mitochondrial *tRNATyr* gene has been described in golden retriever dogs [25], and cardiomyopathy and arrhythmia have been described in a family of Rhodesian ridgeback dogs with a missense variant in the nuclear gene *QIl1* (also called *MICOS13*; OMIA# 001040-9615) [26]. To the author’s knowledge, mitochondrial deletion diseases are rare in canine encephalopathies and not yet reported in canine myopathies.

Here, we describe an adult standard poodle with chronic weakness and exercise intolerance, diagnose a mitochondrial myopathy based on light and electron microscopy of muscle biopsies, and identify multiple deletions in its mtDNA via whole-genome sequencing (WGS) of DNA extracted from muscle biopsies. Insertion of a *DYNLT1* retrogene into the nuclear genome, and downstream alteration of expression of the mitochondrial voltage-dependent anion channel (VDAC), is suspected of being the underlying cause. While not typically evaluated in WGS analyses, this study demonstrates that the evaluation of mtDNA sequences obtained from WGS data can be highly useful in the diagnosis of mitochondrial myopathy.

## 2. Materials and Methods

### 2.1. Clinical Case

An approximately 7-year-old male neutered rescue standard poodle was presented to a board-certified veterinary neurologist at the VCA Animal Care Center of Sonoma County in Rohnert Park, California, USA for evaluation of a 2–3-year history of progressive weakness and exercise intolerance. A neuromuscular disease was suspected and further evaluations including electromyography and muscle biopsies were performed. All studies were performed for diagnostic purposes and with the owner’s consent.

### 2.2. Histopathology, Histochemistry, Immunohistochemistry and Electron Microscopy

Unfixed chilled and formalin-fixed diagnostic muscle and peripheral nerve biopsies were collected from the cranial tibial and triceps muscles, and the common peroneal nerve under general inhalational anesthesia. All biopsies were shipped by an express service under refrigeration to the Comparative Neuromuscular Laboratory, University of California San Diego. Upon receipt, the unfixed muscle biopsies were snap-frozen in isopentane pre-cooled in liquid nitrogen and stored at −80 °C until further processed. Cryosections were evaluated using a standard panel of histochemical stains and reactions including the mitochondrial-specific reactions succinic dehydrogenase (SDH) and cytochrome c oxidase (COX) [27]. Immunofluorescence staining using a monoclonal antibody against mitochondrial voltage-dependent anion channels (1:100, VDAC, Abcam, Boston, MA, USA) was used on case and control cryosections and co-stained with a polyclonal rabbit anti-laminin antibody (1:100, Abcam, Boston, MA; ab11575). Secondary antibodies were goat anti-mouse or goat-anti-rabbit FITC-labeled antibodies from Jackson Immunoresearch, Westgrove, PA, USA. The fixed nerve biopsy was araldite embedded and evaluated in 1 µm resin sections. Formalin-fixed muscle was processed into paraffin by standard procedures, further post-fixed in osmium tetroxide, and dehydrated in serial alcohol solutions and propylene oxide prior to embedding in araldite resin. Sections (1 μm) were stained with toluidine blue for light microscopy and ultrathin sections were stained with uranyl acetate and lead citrate for electron microscopy.

### 2.3. Whole Genome Sequencing and Variant Analysis

DNA was isolated from archived frozen diagnostic muscle biopsy specimens using the Qiagen DNEasy kit according to package instructions. A PCR-free library was prepared from this case and 150 bp paired-end reads were generated on an Illumina HiSeq 4000 sequencer by Azenta Life Sciences (South Plainfield, NJ 07080, USA). For this case, 1.2 billion paired-end reads were generated, corresponding to a mean 70.9-fold genome-wide coverage. Reads were mapped against the dog reference genome assembly (UU Cfam GSD_1.0/canFam4) [28,29] and processed using the OnlyWAG pipeline as described [30]. Raw sequence reads are available in NCBI’s Short Read Archive (submitted on 28 March 2024; SRR28501896) (https://dataview.ncbi.nlm.nih.gov/object/PRJNA937381?reviewer=8q65jbf2t154tm7t96tem3kiv3).

WGS variants from this case were compared to those of control genomes from an internal WGS database developed at the University of Minnesota which contained a total of 671 dogs of 63 diverse breeds including 38 standard poodles. The WGS data from these 671 dogs were processed using the same bioinformatics pipelines referenced above. Variants unique to the affected dog that were within or in close proximity to coding exons were prioritized as high (frame shift, loss or gain of stop or start codon, affecting a splice junction), moderate (missense), or low (synonymous, near splice junction) for further evaluation. A list of the unique coding variants is provided in Table S1. Visual inspection of the entire length of the mtDNA was performed within the integrative genomics viewer (IGV) and compared to a control archived frozen muscle biopsy specimen. Additionally, visual inspection for the presence of the *DYNLT1* retrogene and mtDNA deletions was performed within IGV and compared to an internal collection of 38 standard poodle WGSs. Analysis of retrogene sequence for the presence of promoter and regulatory sites was performed with Promoter 2.0 [31] and TSSG Promoter prediction [32].

## 3. Results

### 3.1. Clinical Case

A neurological examination showed difficulty rising and a stiff, slow gait, worse in the pelvic limbs without ataxia. The dog could only walk 60–80 feet before becoming progressively stiffer and lying down. Cranial nerve examination and postural reactions were normal. Patellar reflexes were absent bilaterally and withdrawal reflexes were reduced in strength in all four limbs. No apparent discomfort was elicited with vertebral column palpation. A neuromuscular disease was suspected and a chronic myopathy was considered most likely. Prior spinal pain could not be ruled out based on the history. The dog was re-evaluated shortly after the initial exam due to becoming acutely unable to stand after a second trip to the groomer. At that time, the dog could only walk about five steps before sitting down and remained weaker in the pelvic limbs; the exam was otherwise unchanged.

Prior to presentation, creatine kinase (CK) activity was elevated (16,585 U/L; reference range 10–200). A recheck of CK activity one week later was 205 U/L and serial CK evaluations remained normal at 305 U/L and 605 U/L, with the latter performed following the trip to the groomer when the dog’s condition had acutely declined. Additional diagnostic testing included vertebral column radiographs showing mild L7-S1 ventral spondylosis, unremarkable routine laboratory evaluations including a CBC and biochemistry panel, normal total T4 (1.9 ug/dL, reference range 1–4), negative *Toxoplasma* serum titers for IgG/IgM, *Neospora* serology, and Leptospirosis PCR, negative tick serology panel with Lyme Quant C6, negative Rocky Mountain Spotted Fever serology, unremarkable abdominal ultrasound and thoracic radiographs with no obvious megaesophagus. The acetylcholine receptor antibody titer was 0.12 nmol/L (reference range < 0.6 nmol/L).

Lumbar MRI showed mild to moderate L7-S1 intervertebral disc herniation and mild contrast enhancement of the caudal L7 endplate consistent with discospondylitis. Lumbar cerebrospinal fluid analysis revealed protein cytologic dissociation (nucleated cell count 2/uL, protein 67.2 mg/dL) and cytology showed a mild increase in the proportion of neutrophils, consistent with possible mild inflammation. Electromyography (EMG) demonstrated prolonged insertional activity in all muscles evaluated and frequent positive sharp waves and fibrillation potentials in the gastrocnemius, quadriceps, triceps, supraspinatus, infraspinatus and cervical paraspinal muscle groups. Biopsies of the peroneal nerve, cranial tibial muscle and triceps muscle were collected in a standard manner under general inhalational anesthesia [33] and submitted to the UCSD Comparative Neuromuscular Laboratory. Pending biopsy results, the dog was treated with oral amoxicillin clavulanate at 16.6 mg/kg PO q12h for suspected discospondylitis. Tramadol was continued at 4 mg/kg PO q8-12 h for pain and meloxicam was discontinued. The dog showed slight improvement and seemed more comfortable. Prednisone was initiated at 0.8 mg/kg/day 1 week later with no notable improvement and was subsequently tapered without clinical decline.

Following receipt of the biopsy results and a diagnosis of mitochondrial myopathy, the dog was again examined approximately 2- weeks later and showed slightly less stiffness in the pelvic limbs, improved ability to rise from recumbency, no signs of discomfort and mild improvement in walking strength, although the dog still tired quickly. Additional diagnostics included resting and post exercise (defined as walking as far as it could and then laying down) serum lactate concentrations which were 8.65 mmol/L and 9.56 mmol/L, respectively (reference range 0.4–2.8). As mitochondrial myopathy was suspected, supplementation with L-carnitine 50 mg/kg PO q12h, Coenzyme Q10 q24h, and vitamin B12 (Cobalequin) q24h was initiated.

At the time of manuscript preparation, approximately 4 months after the initial examination, the dog remains on amoxicillin/clavulanate and the supplements. No signs of discomfort were reported by the owners at home (moaning, kyphosis). Strength is static; the dog is able to walk around the house and outside to urinate and defecate but spends a lot of time lying down and remains reluctant to jump out of the car.

### 3.2. Light and Electron Microscopy

**Figure 1:**
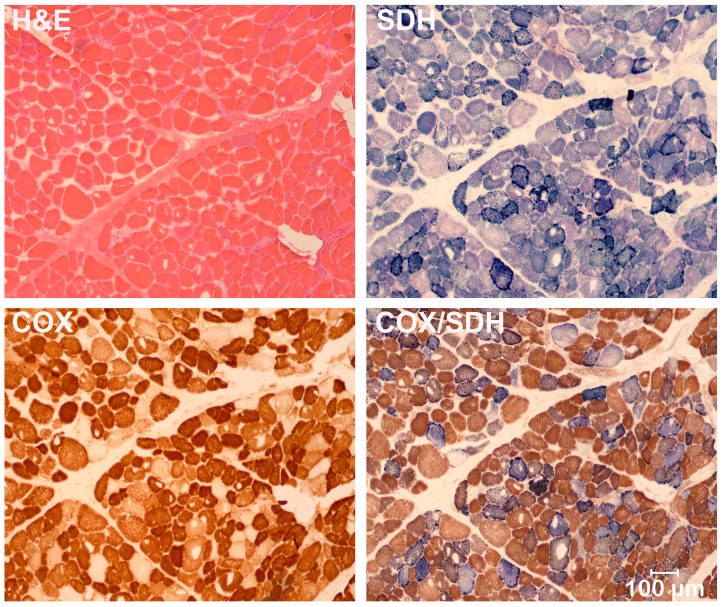
Cryosections from the triceps muscle stained with H&E for general morphology and with the mitochondrial-specific reactions SDH and COX. The staining of all myofibers was present with the SDH reaction and absent in several fibers with the COX reaction. Combined staining for COX and SDH highlights in blue the fibers that are COX-negative.

**Figure 2:**
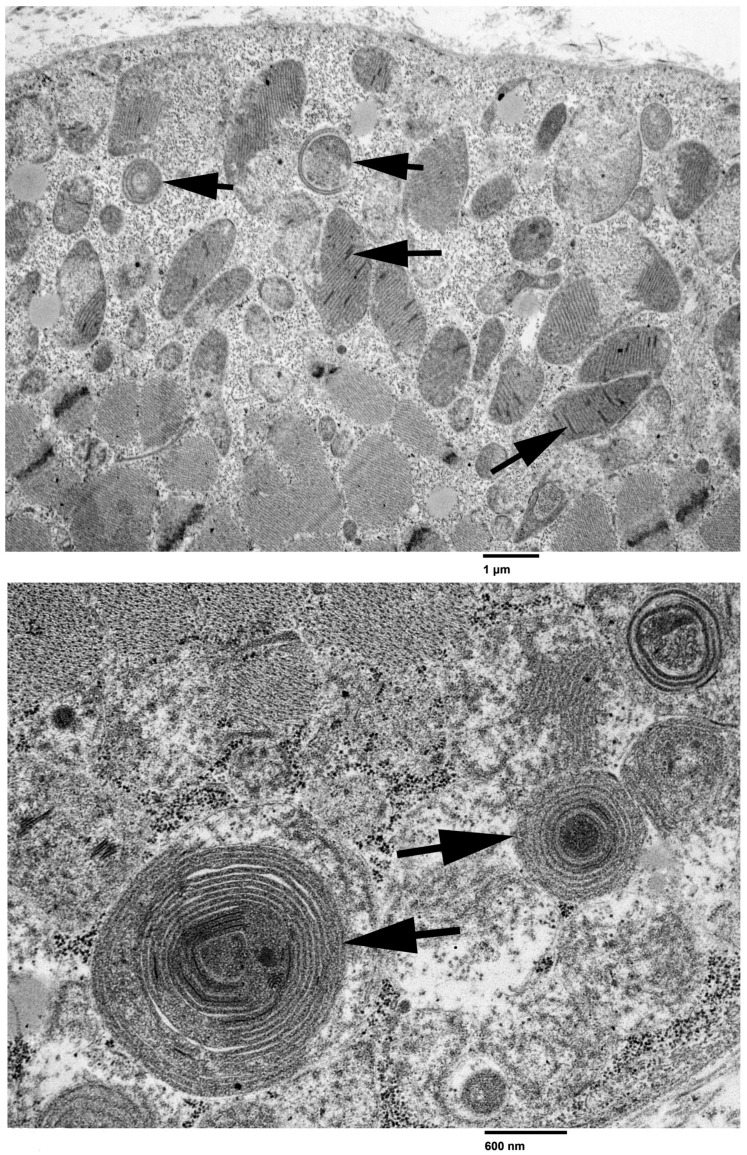
Ultrastructural analysis (upper image low power, bar = 1 µm and lower image high-power, bar = 600 nm) of the triceps muscle. In the low-power image, numerous abnormal mitochondria are shown containing concentrically arrayed (short tail arrows) and linear cristae (paracrystalline inclusions). In the lower high-power image, arrows point to abnormal mitochondria with dense central inclusions surrounded by cylindrical cristae. Mitochondria are surrounded by numerous glycogen granules.

A moderate variability in myofiber size was present in cryosections of the triceps muscle with the H&E stain and several myofibers contained internal dark blue deposits with the SDH mitochondrial-specific reaction. Approximately 36% of the myofibers were COX-negative and stained blue with the combined COX/SDH reaction (Figure 1). Similar changes were present in cryosections from the cranial tibial muscle biopsy (not shown). Ultra-structurally, abnormal mitochondria containing concentrically arrayed tubular cristae and crystalloid inclusions were identified (Figure 2). No abnormalities were identified in the nerve biopsy.

### 3.3. Whole Genome, Mitochondrial DNA Sequencing, and Variant Analysis

**Figure 3:**
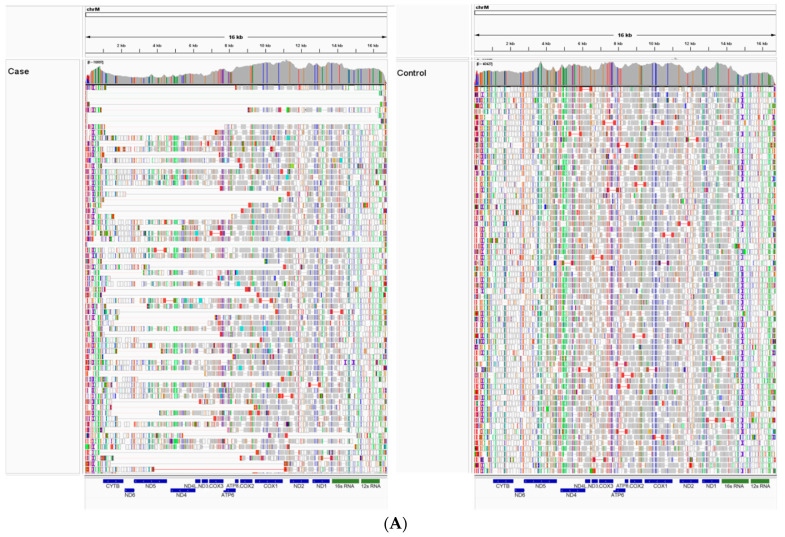

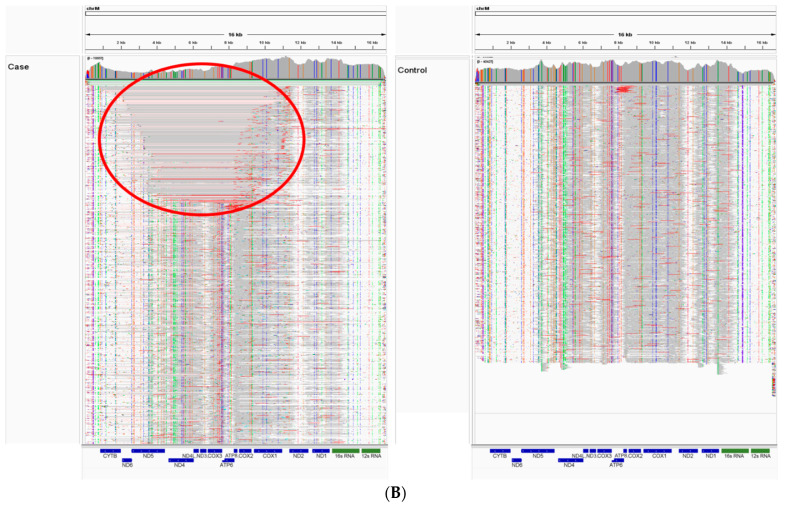
(**A**). Integrative genomics viewer (IGV) expanded view of WGS reads aligned across the ~ 16 kb mitochondrial chromosome. Aligned reads in the case (left) show multiple deletions of various lengths (observed as gaps), in contrast to the contiguous coverage observed in the control (right). The positions of the genes encoded by the mitochondrial genome are indicated in blue rectangles, and the 16S and 12S rRNA genes are indicated in green rectangles. (**B**). Integrative genomics viewer (IGV) “squished” view of WGS reads aligned to the mitochondrial chromosome sorted by insert size. The red circle contains sequence data demonstrating the gaps in coverage and provides an estimate of the percentage of reads that contain deletions.

Genomic DNA extracted from the triceps muscle of the case was submitted for WGS where excellent coverage of both the nDNA and mtDNA was obtained (70.9 fold genome-wide coverage; mtDNA read depth > 18,000). Visual analysis of the mtDNA sequence reads from the case compared to a control French bulldog whose DNA was also derived from skeletal muscle identified numerous deletions that varied in size and were concentrated from approximately chrM:1000–11,000 (Figure 3A) resulting in the full or partial deletion of up to eight mitochondrial genes. Resorting the data alignments according to insert size demonstrated that approximately 25–30% of the mitochondrial genome reads were indicative of a large-scale deletion (Figure 3B).

The phenomenon of multiple mtDNA deletions is often associated with mutations in nuclear-encoded genes involved with mitochondrial maintenance [3]. Analysis of the WGS from the case identified several variants that meet the prioritization criteria for high impact on the encoded gene and were unique to the case (Table S1). All 17 high-impact variants were visually inspected within IGV, and 14 were found to be mapping errors. The three remaining variants included a heterozygous stop codon in *TRANK1*, a heterozygous stop gain point mutation in *POLR2B,* and a retrogene intergenic copy in *DYNLT1* (which was identified as a splice site variant in the VCF due to the cDNA copy). After further investigation, the *TRANK1* gene appeared to have no clear mitochondrial connection. The *POLR2B* gene encodes an RNA polymerase II subunit B component of the cytoplasmic transcriptional machinery that produces both cytoplasmic and most mitochondrial proteins. This stop gained variant results in the truncation of greater than 50% of the POLR2B protein. However, there is no evidence that any other aspect of cellular structure or function is altered as would be expected if the transcriptional apparatus was diminished.

**Figure 4:**
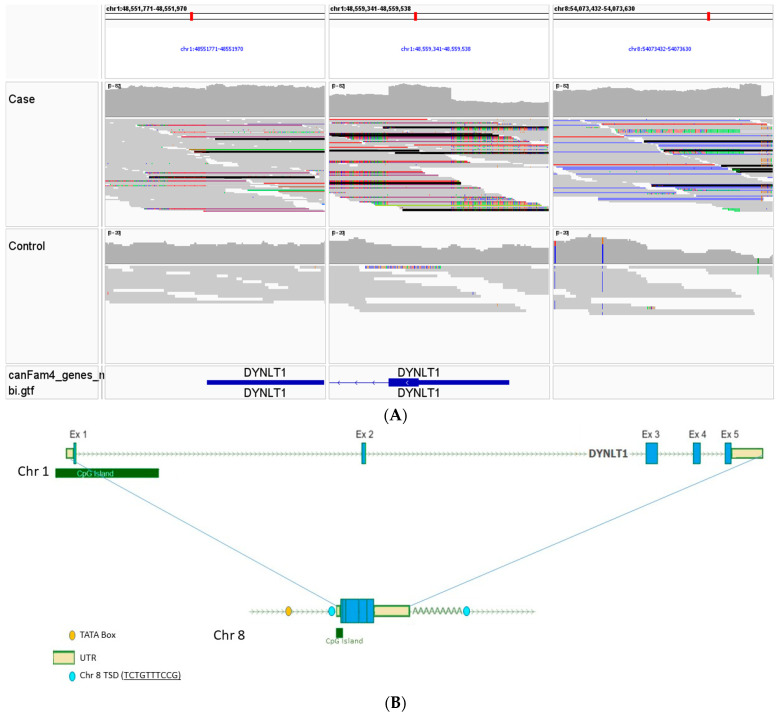

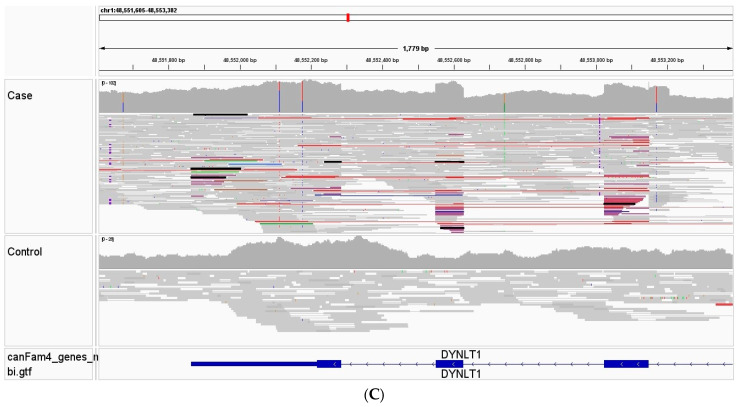
(**A**). WGS reads from a case and control aligned to dog chromosome 1 (**left** and **center**) and chromosome 8 (**right**). The chromosome 1 reads are in the vicinities of the 3′ UTR (left) and 5′UTR (center) of *DYNLT1*, and the chromosome 8 reads are to an intergenic region of chromosome 8. The blue-colored bars in the case dog on the right represent reads that lie in an intergenic region of chromosome 8 in which their mates map to the 3′ UTR or 5′ UTR (clusters of colored reads) of *DYNLT1* on chromosome 1. (**B**). Diagram of the parental *DYNLT1* gene on chromosome 1 (top) and retrotransposition of its cDNA to chromosome 8 (bottom) at position chr8:54,073,620 of the CanFam4/UU_Cfam_GSD_1.0 reference genome. Shown on the parental gene are, from left to right, CpG sequence, the 5′ UTR, 5 exons and the 3′ UTR. Shown on the retrogene are a predicted TATA box at chr8:54,073,513-54,073,518 with potential transcriptional start sites predicted within the upstream TSD sequence or 5 bp upstream of the start codon, along with 30 bp of the 5′ UTR, all of the 3″ UTR, a poly A tail and the TSD sequences. (**C**). Evidence for the presence of a *DYNLT1* retrogene in the case dog. The red mate pairs aligned over consecutive exons of *DYNLT1* (skipping over intronic sequence but connecting red lines indicating their mates) are expected from reads derived from a retrogene-mediated *DYNLT1* cDNA insertion elsewhere in the genome. A similar result is not observed in the control dog.

**Figure 5:**
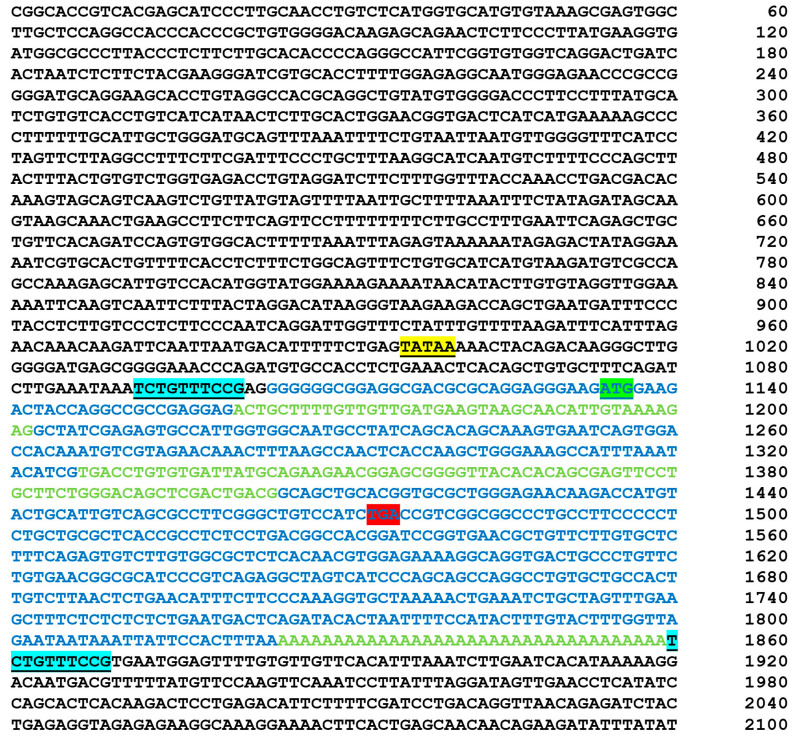
Sequence and annotation of the *DYNLT1* retrogene. The TSD sequences on either end of the insert sequence are underlined and highlighted in blue, the inserted *DYNLT1* cDNA sequence is in blue and green fonts with alternating exons switching color, the ATG start codon at position 1129 is highlighted in green, and the TGA stop codon is highlighted in red. The insertion site starts at position 54,073,620 on canine chromosome 8.

The third variant is a heterozygous intergenic retrogene insertion (chr8: 54,073,620; Figure 4A–C) of *DYNLT1*, a regulator of the voltage-dependent anion channel (VDAC), that plays an important role in mitochondrial function. This retrogene is derived from the insertion of processed *DYNLT1* cDNA from the parent *DYNLT1* gene on chromosome 1 and is indicated by WGS read mate pairs in the case aligning to both chromosomes 1 and 8. Detailed analysis of the *DYNLT1* retrogenic sequence identified the target-site duplication (TSD) sequences necessary for insertion, as well as sequences enabling the potential for serving as a template for transcription and expression, including a TATA box, promoter, and CpG island (Figure 5).

### 3.4. Immunohistochemical Staining

**Figure 6:**
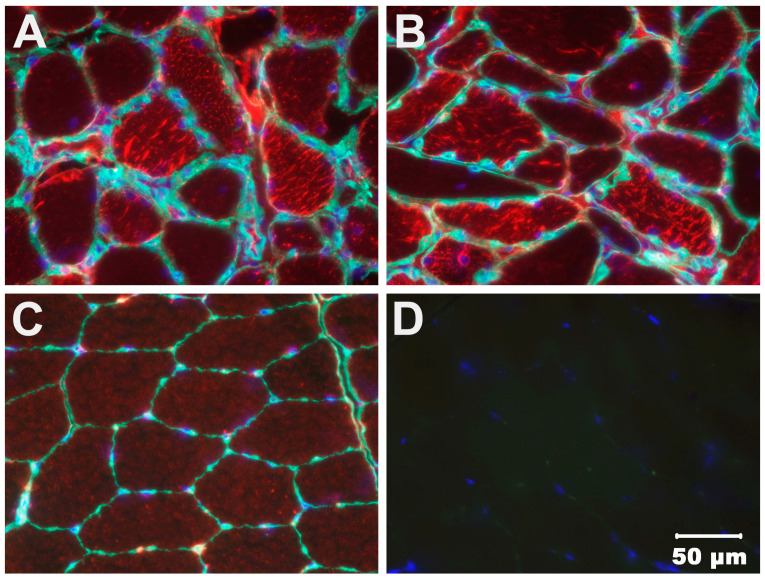
Immunohistochemical staining of cryosections from the cranial tibial muscle (**A**) and triceps muscle (**B**) from the affected dog and the triceps muscle from an archived adult control dog (**C**) using a monoclonal antibody against voltage-dependent anion channels (VDAC, red stain) and an antibody against laminin (green stain). Dapi stain (blue) indicates muscle nuclei. The image in (**D**) is a second antibody only. The increased staining with the antibody against VDAC in the affected dog muscles and only faint staining in the control dog muscle suggests the retrogene variant in the affected dog is active with an increased level of expression.

We considered it possible that the retrogenic insertion of a *DYNLT1* cDNA in the case could potentially be transcribed and in turn alter the level of expression of this regulator of the mitochondrial voltage-dependent anion channel (VDAC, porin). To test this hypothesis immunostaining of case and control muscle cryosections was performed using a monoclonal VDAC antibody (anti-VDAC1, 1:100, ab14734, Abcam). Compared to the control muscle, the staining of muscle from the clinical case was increased (Figure 6).

## 4. Discussion

Mitochondria are present in all nucleated cell types; thus, mitochondrial diseases can affect any organ or tissue in the body. Clinical presentation may be as an organ-specific disease, such as myopathy, cardiomyopathy, or optic neuropathy, or a systemic disease. Mitochondrial myopathies are an important group of progressive muscle diseases, caused primarily by the impairment of oxidative phosphorylation (OXPHOS). In fact, myopathy is one of the most common manifestations of adult-onset mtD due to the high cellular energy demand of skeletal muscle [3]. Here, we describe an adult dog with clinical signs of chronic exercise intolerance and resting and post-exercise lactic acidemia without evidence of behavioral changes or cardiomyopathy. It is difficult to reach a diagnosis of a specific myopathy based on clinical presentation alone as many congenital myopathies in dogs can present with signs of exercise intolerance, weakness, stiffness and gait abnormalities [34]. The diagnosis of mitochondrial myopathies therefore should include a multidisciplinary approach including histological, immunohistochemical assays, electron microscopic studies, enzymatic analysis of OXPHOS complexes, and genetic analysis using WGS.

Histological examination of skeletal muscle biopsies by a neuromuscular pathologist using specific histologic and histochemical stains and reactions is important for ruling out other congenital, dystrophic and acquired myopathies and obtaining a correct diagnosis. Staining of muscle cryosections with the modified Gomori trichrome stain can identify the presence of ragged-red fibers. Ragged-blue fibers can be detected using the SDH reaction (SDH, complex II). Both reactions detect mitochondrial aggregates in the subsarcolemmal region of the muscle fiber due to mitochondrial proliferation that occurs with mitochondrial OXPHOS dysfunction [27]. Another important diagnostic finding is the presence of cytochrome c oxidase (COX, complex IV) negative fibers detected by sequential COX/SDH histochemistry (Figure 1). A mosaic pattern is commonly observed with COX-negative fibers appearing blue, while the normal COX-positive fibers appear brown. The mosaic pattern is due to different levels of heteroplasmy with a high mutation load leading to respiratory chain deficiency. As aging individuals can accumulate a low frequency of COX-negative fibers, a diagnosis of mitochondrial myopathy is only made when individuals harbor COX-negative fibers at a frequency of >5% [35,36]. In our case, 36% of muscle fibers were COX-negative supporting a clear diagnosis of mitochondrial myopathy. Ultrastructural evaluation of muscle confirmed the presence of abnormal appearing mitochondria and paracrystalline inclusions. The determination of serum lactate concentrations at rest and following exercise with documentation of lactic acidemia has also become a diagnostic tool for mitochondrial myopathy [37].

WGS was performed to analyze both the nuclear and mitochondrial DNA genomes for the presence of candidate functional variants consistent with the diagnosis of mitochondrial myopathy. In addition to the identification of potentially causative variants in nuclear genes, WGS also allows the detection of alterations in the mitochondrial genome including low levels of heteroplasmy, point mutations, and breakpoints of single, large-scale and multiple mtDNA deletions [3]. This enabled the identification of multiple mtDNA deletions in this clinical case compared to a control genome (Figure 3A,B). The presence of multiple mtDNA deletions in muscle is often a hallmark of mitochondrial myopathy, particularly those caused by mutations in nuclear genes encoding proteins required for mtDNA homeostasis, replication and maintenance such as *POLG*, *POLG2* and *TWNK*, the mitochondrial polymerase and helicase [38]. No unique coding or structural *POLG* or *TWNK* variants were identified in our case. Three other variants were considered to have a high negative impact using protein predictors and were not identified in a large canine control population in the University of Minnesota database including 38 standard poodles. These included a heterozygous stop codon in *TRANK1*, a heterozygous stop gain point mutation in *POLR2B*, and a retrogene intergenic copy in *DYNLT1.* After further investigation, the *TRANK1* gene appeared to have no clear mitochondrial connection. Although *POLR2B* is an RNA polymerase II, alterations in RNA translational proteins are thought to result in decreased quantities of cellular mRNA and protein expression but do not affect mtDNA replication or repair. *POLR2B* is also not reported in OMIM (180661, https://www.omim.org/) or in the NIH GeneCards [https://www.genecards.org/cgi-bin/carddisp.pl?gene=POLR2B assessed on 20 April 2024] as making a mitochondrial protein, although the human protein atlas suggests POLR2B is mainly mitochondrial (https://www.proteinatlas.org/ENSG00000047315-POLR2B/tissue assessed on 20 April 2024). Thus, clear causality for *POLR2B* in this mitochondrial myopathy could not be established.

While not thought to be relevant to mtDNA maintenance or repair, the *DYNLT1* gene encodes an important regulator of the voltage-dependent anion channel VDAC, a gated porin that permits the transport of peptides, metabolites and ions [39,40,41,42]. We considered the hypothesis that an alteration in *DYNLT1*, with subsequent effects on increasing VDAC expression, could be an important event underlying the pathogenesis of this mtD. VDAC plays an important role in mitochondrial processes such as signaling, apoptosis, and calcium homeostasis and all three isoforms reported in humans are present at high levels in the heart, kidney, skeletal muscle and brain [43]. VDACs form pores in the outer mitochondrial membrane capable of metabolite transport. The gating of VDAC includes open, closed, multimeric, and blockage of the pore by specific proteins and metabolites. When these interactions are disrupted, they lead to disease states including Fredreich’s ataxia and Parkinson’s disease [44]. VDAC channels are permeable to calcium from the cytosol and an increase in flux of calcium into the mitochondria is known to lead to apoptosis [44].

Increased VDAC expression in the case was demonstrated by immunostaining of muscle cryosections. This is consistent with a *DYNLT1*-mediated process and could potentially be a result of overexpression or atypical of *DYNLT1* through the retrogene insertion of its cDNA into a transcriptionally active form. Although we have no direct evidence to show that the *DYNLT1* retrogene is expressed, its cDNA sequence is identical to that derived from the nuclear gene, so if transcribed it could presumably be translated into a functional protein. Work by others suggests such retrogenes can be active by borrowing contextual regulatory elements such as upstream promoter sequences, or enhanced by a CpG island, to promote expression of the retrogene with control provided by the 3” UTR [45]. In addition to the lack of additional frozen tissue to assess *DYNLT1* gene expression, other limitations to this study include the absence of DNA from parents of the case or other family members as the dog was obtained as a rescue. Thus, we are unable to tell if this was an inherited condition or a spontaneous mutation.

## 5. Conclusions

A mitochondrial myopathy based on a histological and histochemical phenotype was determined to be the cause of exercise intolerance and lactic acidemia in our adult dog. WGS established the presence of multiple mtDNA deletions suggesting an underlying nuclear DNA variant affecting mitochondrial replication, repair or metabolism. The significance of the *DYNLT1* retrogene as a functional candidate is not entirely clear but presents an intriguing possibility through its links to the VDAC and overall mitochondrial function. Although beyond the scope of this case report, functional studies in cell lines (fibroblast culture) to investigate protein and RNA analyses, protein transport and import, and transcription and mtDNA maintenance, and model systems (yeast, drosophila, zebrafish or mouse) are necessary to prove causality.

## Data Availability

Raw sequence reads are available in NCBI’s Short Read Archive (SRR28501896) under BioProject PRJNA937381. Permanent link provided following publication.

## Supplementary Materials

The following supporting information can be downloaded at: https://www.mdpi.com/article/10.3390/ani14131946/s1, Table S1; List of unique coding variants.
